# Augmented Reality and Learning-Cognitive Outcomes in Autism Spectrum Disorder: A Systematic Review

**DOI:** 10.3390/children12040493

**Published:** 2025-04-10

**Authors:** Cristina Fuentes, Soledad Gómez, Simona De Stasio, Carmen Berenguer

**Affiliations:** 1 Campus Capacitas, Catholic University of Valencia, 46002 Valencia, Spain; cf.saez@mail.ucv.es (C.F.); soledad.gomez@ucv.es (S.G.); 2 Department of Human Studies, LUMSA University, 00193 Rome, Italy; s.destasio@lumsa.it; 3 Department of Developmental and Educational Psychology, University of Valencia, Avda. Blasco Ibáñez, 21, 46010 Valencia, Spain

**Keywords:** autism, augmented reality, technology, learning-cognitive outcomes

## Abstract

Background/Objectives: Augmented reality (AR) has emerged as a promising educational tool for individuals with autism spectrum disorder (ASD), offering interactive and engaging learning experiences. While AR interventions have been widely explored in educational contexts, their specific impact on learning outcomes in individuals with ASD remains unclear. This systematic review aimed to explore preliminary indications of the efficacy of augmented reality (AR)-based interventions in improving cognitive and academic skills in children, adolescents, and adults with ASD. Methods: A comprehensive literature search identified studies published between 2014 and 2024 that assessed AR interventions targeting learning outcomes in individuals with ASD. Results: A total of 12 studies (9 were single-subject studies), comprising 123 participants, met the inclusion criteria. The findings revealed that AR interventions contributed to improvements in multiple learning domains, including language acquisition, reading comprehension, mathematics, science education, executive functioning, and attention. AR-based applications were particularly effective in enhancing engagement, motivation, and interactive learning, addressing challenges commonly faced by individuals with ASD. Conclusions: Findings suggest that AR can be a valuable tool for improving learning outcomes in individuals with ASD, and it could contribute to the inclusion and functional development of students with special needs.

## 1. Introduction

Autism spectrum disorder (ASD) is a chronic neurodevelopmental disorder primarily characterized by deficits in social communication and restricted, repetitive, and stereotyped patterns of behaviors (American Psychiatric Association, APA [1]). At the same time, the prevalence of ASD remains high among children and continues to rise significantly [2]. Current estimates suggest that approximately 1 in 100 children worldwide are affected by ASD [3], positioning it as a major public health concern with substantial economic implications [4]. Given this context, implementing interventions for children, adolescents and adults with ASD is a logical approach, as early intervention can foster greater independence and potentially reduce long-term support costs.

Augmented reality (AR) has gained attention as a promising educational tool, enhancing teaching and learning by integrating virtual elements into real-world environments. AR creates immersive, interactive, and engaging learning experiences that may be challenging to replicate in traditional settings [5]. Unlike Virtual Reality (VR), which immerses users entirely in digital environments, AR overlays digital content onto the physical world, providing multimodal learning experiences that cater to diverse learner profiles, including those with ASD [6].

Despite the growing interest in AR-based educational interventions, research on its application for individuals with ASD remains fragmented. While previous studies have explored AR’s role in education, they often focus on broader aspects such as behavioral and social skills rather than its direct impact on learning outcomes [7]. Additionally, many existing reviews lack methodological rigor, limiting their ability to provide a comprehensive and evidence-based evaluation of AR’s educational benefits for autistic individuals [8].

A growing body of research has highlighted AR’s potential to improve social, cognitive, and academic skills in individuals with ASD [9,10]. Studies indicate that AR facilitates the comprehension of abstract concepts, enhances task sequencing, supports visual scheduling, and improves adaptability to routine changes—key challenges for autistic learners [11,12]. In the same line, Howorth et al. [13] focused on literacy development, describing AR applications to support reading activities for students with ASD, highlighting the potential benefits.

Moreover, AR applications incorporating gamification elements have been shown to increase engagement and motivation, encouraging active participation in learning activities. Some studies have demonstrated AR’s effectiveness in teaching practical life skills such as toothbrushing, emotion recognition, and socialization [14,15] as well as its role in vocabulary acquisition, reading comprehension, and science instruction [16,17].

However, existing reviews on AR-based interventions for autistic individuals exhibit significant variability in methodologies, sample sizes, and study designs, leading to inconsistencies in reported outcomes. For instance, El Shemy et al. [18] conducted a thematic analysis of AR-enhanced language learning applications, incorporating empirical and observational studies. Their findings identified 13 studies that focused on language acquisition and 31 that focused on language use, revealing that AR significantly improved vocabulary retention and communication skills in children with ASD. However, a limitation of this review was the absence of standardized assessment tools for evaluating the effectiveness of AR language applications. This variability in assessment methods makes it challenging to compare findings across studies and establish generalizable conclusions.

Similarly, Khowaja et al. [19] reviewed over 55 studies, from the years 2005 to 2018, categorizing AR interventions based on their impact on cognitive, social, and behavioral skills. Their results indicated that AR-based learning tools positively influenced cognitive and behavioral outcomes, though the effectiveness varied depending on individual needs and the nature of the interventions. The study also highlighted the need for adaptive AR applications tailored to individual learning preferences. A limitation of this review was the absence of studies that assessed the impact of AR over an extended period.

Another review conducted by Fridhi and Bali [20] brought a reflection on the design and learning methodology in the field of adapted physical activities in children with ASD. However, they did not conduct a systematic review, so their conclusions cannot be generalized.

Lorenzo et al. [21] adopted a longitudinal approach, tracking AR applications over a two-decade period and categorizing the evolution of AR-based educational tools. Analyzing 28 studies, they found that AR has significantly evolved in terms of technology, accessibility, and pedagogical integration. They emphasized the importance of designing AR interventions that align with individualized education plans for autistic learners. However, they conducted a thematic review.

Finally, Yakubova et al. [22] included eight peer-reviewed studies, published between 2007 and 2020, and targeted mostly literacy (n = 3), science (n = 2), mathematics (n = 2), and collateral academic skills (n = 1) to assess AR’s role in teaching core subjects such as mathematics, science, and reading comprehension in children with ASD and children with developmental disabilities. Their analysis suggested that AR can be highly effective in delivering academic instruction, particularly when integrated with structured teaching methodologies. However, their review included individuals with ASD and/or with intellectual disability as a primary diagnosis, and it did not examine the effects of AR interventions on adults.

Given these gaps, there is a critical need for a systematic review that rigorously examines the impact of AR on learning outcomes in individuals with ASD.

The objective of this systematic review is to investigate the impact of augmented reality (AR) on learning outcomes and cognitive processes in children, adolescents, and adults with autism spectrum disorder (ASD), with or without intellectual disability. Specifically, this review examines how AR-based interventions contribute to improvements in both academic skills (such as science, language acquisition, reading, and mathematics) and cognitive-attentional skills, including attention and executive functions. Additionally, we aim to analyze the extent to which these interventions align with evidence-based quality criteria, as proposed by Reichow [23]. Given the growing body of research in this field, we consider that AR applications provide additional opportunities for implementing evidence-based treatments that enhance essential cognitive and learning skills, ultimately supporting functional adaptation in individuals with ASD.

To the best of our knowledge, this investigation represents the first systematic review dedicated to evaluating the use of AR technologies for enhancing learning outcomes in individuals with ASD. By identifying gaps and methodological limitations in existing research, this study will contribute to advancing knowledge in the field and guiding future interventions aimed at improving educational opportunities for autistic individuals.

## 2. Materials and Methods

This systematic review and meta-analyses were developed in accordance with the criteria of the “Preferred Reporting Items for Systematic Reviews and Meta-Analysis” (PRISMA) [24].

### 2.1. Eligibility Criteria

The eligibility criteria utilized in the process of preliminary screening and full-text screening were developed in accordance with the population (P), intervention (I), comparison (C), and outcome(s) (O) model [25] as detailed below:

P Population—Individuals with ASD.
I Intervention—AR-based treatment.
C Comparison—Non-AR-based treatment or treatment as usual.
O Outcomes—Learning outcomes obtained.

Furthermore, additional inclusion criteria were implemented: (i) included at least one individual with ASD of 3–40 years with autism spectrum disorder identified according to ASD diagnostic criteria, such as those in the DSM; (ii) studies that use AR interventions; (iii) studies that have improving executive functions or learning outcomes as the main objective of the intervention; (iv) quasi-experimental, group comparison, experimental, or single-case experimental design; (v) empirical studies (research approach that involves the systematic collection and analysis of data to investigate a specific research question or hypothesis, based on direct or indirect observation or experience); (vi) studies published in peer-reviewed journals in English; and (vii) studies published between 2014 and 2024. Exclusion criteria were as follows: (i) if the sample was not clearly identified as individuals with ASD; (ii) not clearly reported intervention outcomes; (iii) qualitative methods, mix methods; (iv) studies not published in peer-reviewed journals in English; (v) studies not published between 2014 and 2024; and (vi) reviews, dissertations, reports, congress communications, and proceedings were excluded.

### 2.2. Search Strategy and Data Screening

The systematic search was conducted in October 2024 to collect the pertinent literature from reliable electronic databases, including PsycINFO, Education Resources Information Centre (ERIC), PubMed, Scopus, Web of Science Core Collection, Scopus, Psychology, and Behavioural Sciences Collection (EBSCO). These databases were selected since they each cover a large part of the relevant literature on these topics. The keywords were combined to search the literature by using the Boolean operators AND/OR: (Augmented reality OR AR) AND (autism* OR ASD* spectrum disorder) AND (learning skills OR educat* OR cognitive OR attention OR memory) AND (treatment OR intervention OR train* OR program OR remed*). The truncations were specifically employed to ensure the retrieval of every possible variation in the chosen keywords. The references of each study that met the inclusion criteria were examined. Forward citations were reviewed, and the surnames of the first authors were searched in Google Scholar to identify additional relevant publications. Finally, the references of three reviews were hand-searched [18,19,22]. All relevant articles were extracted from the ancestral and citation search into a Microsoft Excel ™ spreadsheet.

Two authors independently searched the literature and reviewed all the studies. All authors screened the articles for inclusion and evaluated the evidence level using Reichow’s [23] assessment framework. The screening process consisted of a title and abstract screening as well as a full-text screening based on the PICO criteria. If a study met all the predefined eligibility criteria, it was included in this review. A total of 90% initial agreement was achieved on the database search. Any division of opinions was resolved by consensus among the authors of this work. This process was repeated until 100% agreement on the inputted data was reached.

### 2.3. Methodological Quality Evaluation

The methodological rigor of the studies included in this systematic review was evaluated using Reichow’s [23] assessment framework. This methodology was specifically designed to assess empirical research on targeted interventions for individuals with autism spectrum disorder (ASD) and was applicable to both single-subject and group comparison research designs [9,22]. The evaluation process followed a structured three-phase protocol to ensure methodological consistency and reliability. In the initial phase, the quality of each study was assessed based on primary quality indicators. These indicators included a comprehensive characterization of study participants, precise operational definitions of independent and dependent variables, the establishment of baseline conditions, and the application of visual data analysis techniques. Additionally, secondary quality indicators were considered, including interobserver agreement, the involvement of blinded raters, computation of the Kappa coefficient, intervention fidelity, assessment of generalization or maintenance of treatment effects, and evaluation of social validity. While these secondary indicators contributed to the overall methodological rigor, they were not deemed essential for determining study validity. Each study received a quality rating of “high” (H), “acceptable” (A), or “unacceptable” (U) based on these indicators.

Following the initial assessment, studies were categorized into three levels—“strong”, “adequate”, or “weak”—based on their cumulative quality ratings. In the final phase, studies were aggregated according to two primary criteria: the number of participants in single-subject research designs and the number of group comparison studies demonstrating effective intervention outcomes within studies classified as either “strong” or “adequate”. The above evidence-based practice (EBP) formula was applied, per intervention, to determine all possible combinations of evidence: (Group_S_*30) + (Group_A_*15) + (SCRD_S_*4) + (SCRD_A_*2) = Z. Group_S_ is the number of studies conducted using group research designs with a strong level of evidence, Group_A_ is the number of studies conducted using group research designs with an adequate rating, SCRDs indicates the number of participants for whom the intervention was successful from SCRD studies with a strong rating, SCRD_A_ is the number of participants for whom the intervention was successful from SCRDs studies with an adequate level of evidence rating, and Z is the total number of points for each study with 60 points indicating “established (EBP)” and >30 points indicating “probable EBP”.

## 3. Results

### 3.1. Literature Search

**Figure 1:**
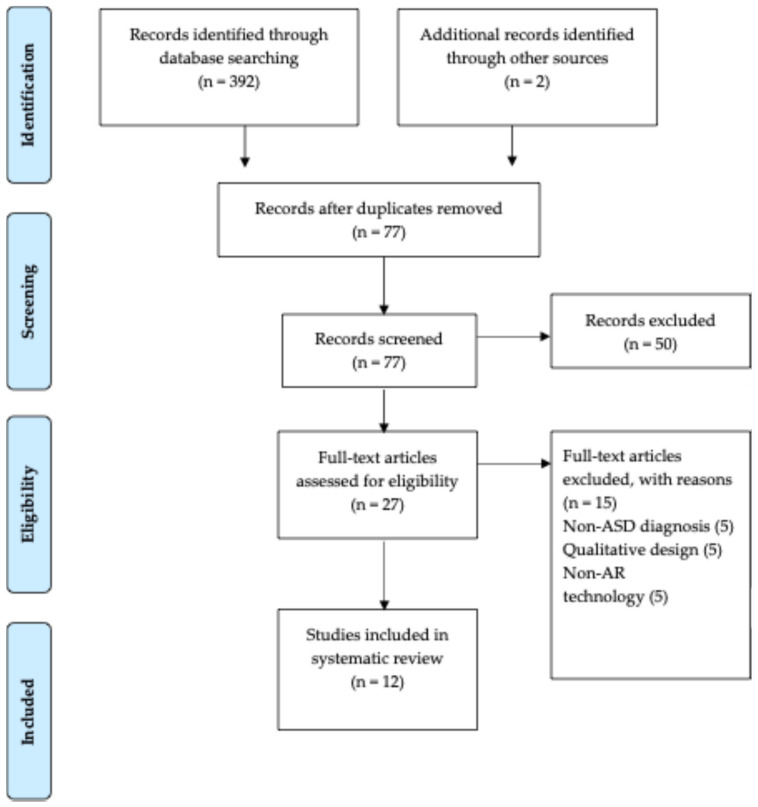
PRISMA flowchart of study selection process for this systematic review.

The literature search yielded 394 articles distributed over time as shown in Figure 1.

After the removal of duplicates, 77 titles remained. Of these, 50 studies were excluded after the title and abstract screening, as they did not fulfill the eligibility criteria. Of the remaining 27 studies that were subjected to the full-text screening, 15 did not meet the inclusion/exclusion criteria. Specifically, five studies were excluded as they did not report an ASD diagnostic evaluation with a validated measure, five studies were excluded as they had qualitative design, and, finally, five studies were excluded as they included virtual technology that were not AR technology. Finally, 12 studies met the eligibility criteria and were included in this systematic review. The characteristics of the 12 studies are summarized in Table 1.

### 3.2. Study Characteristics

All the included studies were empirical studies analyzing the effect of augmented reality-based interventions to improve learning outcomes in children, adolescents, and adults with ASD. They were published in English language (see Table 1).

The total number of participants with ASD in the included publications was 123. The gender distribution of the participants in this sample was male n = 89 and female n = 12, meaning that 11.88% of participants were female, which is consistent with the reported male-to-female gender ratio of approximately 3:1 in ASD [37]. However, the reported number of male–female participants in this total sample may be inaccurate due to two of the studies [31,33] not reporting gender distribution. Furthermore, ten studies reported a clinical sample with an IQ >70 [16,26,27,29,30,31,32,33,35,36], and two studies reported a clinical sample with a low IQ (<70) [28,34]. Most studies reported samples of children and adolescents with ASD, and only two studies included adults with ASD [28,35].

All studies were published from 2014 to 2024 and conducted in eight countries: United States (n = 3) [28,35,36], Turkey (n = 2) [26,27], Malaysia (n = 2) [16,31], Indonesia (n = 1) [29], Brazil (n = 1) [30], Republic of Korea (n = 1) [32], Mexico (n = 1) [33], and Sapin (n = 1) [34].

This review analyzed nine single-subject design studies [16,26,27,28,29,33,34,35,36]: one utilized a non-randomized group design [30], one implemented a within-subject design using two conditions (AR mobile learning platform *versus* conventional teaching) [31], and one implemented a randomized controlled trial with a pretest–posttest design [32]. All reviewed studies incorporated augmented reality (AR) in their intervention processes.

In terms of technology, seven studies (58.4%) implemented AR applications using smartphones or mobile devices. Denizli-Gulboy et al. [26] utilized the *Vucudumuz 4D* AR mobile application and AR cards with a tablet. Gulboy et al. [27] applied the *Octaland 4D* AR application to teach occupations, featuring 26 different 4D occupational flashcards. Hashim et al. [16] employed the *AReal-Vocab* mobile application to enhance English vocabulary learning. McMahon et al. [28] used *Aurasma*, an AR app that enables users to create and interact with customized digital content overlaid on printed markers, with instructional videos designed for teaching vocabulary. Khoirunnisa et al. [29] developed a mobile AR application using the Linear Sequential Model, allowing students to independently select vocabulary words to learn through an interactive SAS reading method. Mahayuddin et al. [31] implemented a prototype Android application utilizing AR marker-based technology through the Vuforia platform, incorporating AR phonics learning and 3D educational games. Escobedo et al. [33] developed *Mobis*, a smartphone AR application that supports the direct annotation of digital content, including text, audio recordings, and visual overlays on physical objects.

The remaining five studies (41.6%) employed AR applications via desktops or tablets. Antão et al. [30] used *MoviLetrando*, a desktop-based interactive learning program that projected virtual symbols such as letters and numbers onto a screen. Nekar et al. [32] implemented a desktop AR game-based cognitive–motor training system. Pérez-Fuster et al. [34] employed *The Pictogram Room*, a suite of video games designed to train various cognitive and motor skills, such as body knowledge and joint attention, using a personal computer (PC), Kinect for Xbox, and additional audiovisual recording equipment. Root et al. [35] utilized AR-based video instruction for personal finance problem-solving skills, where students accessed instructional videos through the HP Reveal app by scanning AR markers. Similarly, Morris et al. [36] integrated AR through the HP Reveal app on an iPad, supplementing instruction with worksheets designed using Math Resource Studio 6 by Schoolhouse Technologies, enabling students to scan embedded images and access corresponding instructional videos.

### 3.3. Main Results

All studies reviewed reported that the application of augmented reality (AR)-based interventions led to improvements in at least one of the targeted learning outcomes (see Table 2). The primary focus of these studies was the enhancement of learning outcomes and cognitive processes, particularly those related to attention and executive functions. The reviewed research explored AR’s effects across various educational domains, including science, language acquisition, reading, mathematics, and cognitive-attentional skills.

Denizli-Gulboy et al. [26] and McMahon et al. [28] examined the use of AR in science education. Denizli-Gulboy et al. [26] aimed to teach students the names of internal organs from different body systems (digestive, circulatory, respiratory, and excretory) in accordance with the national science curriculum guidelines. Results indicated that systematic instruction effectively facilitated navigation of the AR application and organ naming. Similarly, McMahon et al. [28] investigated the effects of marker-based AR technology in teaching science-related vocabulary to college students with intellectual disabilities (IDs) and ASD. Visual analysis confirmed that AR-based instruction was an effective strategy for enhancing science vocabulary acquisition.

Two other studies assessed AR’s impact on language and vocabulary learning. Gulboy et al. [27] evaluated the effectiveness of AR integrated into the SGIA (Systematic Generalization Instructional Approach) for teaching occupational names and related tasks to children with ASD. The study found that AR-supported instruction facilitated target skill acquisition, maintenance, and generalization. Similarly, Hashim et al. [16] developed the AReal-Vocab AR mobile application, which successfully enhanced pronunciation skills and language articulation in children with moderate ASD. The study emphasized that the interactive and visually stimulating 3D elements in AR captivated children’s attention and encouraged language development at home.

Two studies focused on AR’s impact on reading skills. Khoirunoisa et al. [29] demonstrated that personalized AR applications significantly improved ASD students’ ability to read words and syllables from baseline to final assessment. These findings suggest that AR can make early reading instruction more engaging and interactive, supporting previous research [13,28]. Likewise, Mahayuddin et al. [31] evaluated an AR mobile application for phonics literacy in autistic children, reporting positive responses and improvements in phonics learning.

The effects of AR on executive function (EF) and reaction time in ASD learning processes were examined in two studies. Antão et al. [30] found that individuals with ASD were able to play an AR game involving letters and numbers, with observed improvements in reaction time following task completion. Similarly, Nekar et al. [32] investigated AR-based cognitive–motor training’s effects on different EF components, including working memory, cognitive flexibility, and cognitive inhibition. The findings indicated significant improvements in EF accuracy and reaction time after AR-based interventions.

Two studies explored AR’s role in enhancing attention skills. Escobedo et al. [33] found that Mobis, an AR application, increased students’ on-task time by 20%, thereby improving attention skills that are crucial for learning. Meanwhile, Pérez-Fuster et al. [34] focused on joint attention (JA), a critical skill for learning that involves shared focus via eye gazing, pointing, or verbal/non-verbal cues. The study demonstrated that AR technology effectively improved JA in six children, enabling them to follow gaze cues and direct attention appropriately. Post-intervention, all participants generalized their JA skills to interactions with researchers, reinforcing AR’s potential in fostering attention-related competencies.

Finally, two studies investigated the impact of AR on mathematics learning in four young adults and one adolescent with ASD. Root et al. [35] examined a multicomponent intervention that included video-based AR instruction to enhance personal finance problem-solving skills. The study found a functional relationship between AR-based interventions and improvements in financial problem-solving abilities. Additionally, participants successfully generalized these skills to new contexts with minimal support. Similarly, Morris et al. [36] evaluated the use of AR technology in teaching targeted mathematics skills to one adolescent with ASD. The results indicated significant improvements in mathematical skills, with high levels of skill retention post-intervention.

### 3.4. Methodological Quality Evaluation

The research strength of all included studies was calculated in accordance with Reichow’s [23] criteria. Table 3 provides a summary of the strength ratings for each study included in this systematic review. Seven studies (58.33%) received an adequate rating [26,27,29,30,34,35,36], three studies (25%) received a weak rating [16,31,33], and two of the included studies (16.66%) that used an AR intervention were rated as strong [28,32]. The evidence base for augmented reality interventions was calculated. This yielded a Z score of 56, indicating that augmented reality interventions could be categorized as probable in evidence-based practice [23].

## 4. Discussion

The objective of this systematic review was to examine the impact of AR on cognitive processes and learning outcomes in children, adolescents, and adults with ASD. Specifically, this review investigated how AR-based interventions contributed to the improvement of academic skills—such as language acquisition, mathematics, reading, and science—as well as cognitive–attentional abilities, including executive functions and attention. We identified 12 studies, all of which targeted some learning skills such as science, language/vocabulary, reading, reaction time/executive functions, attention processes, and mathematical learning.

The findings reveal that two of the twelve studies investigating the effect of AR interventions on learning outcomes in individuals with ASD are strong [28,32], seven are empirically adequate [26,27,29,30,34,35,36], and two are empirically weak in rigor rating [31,33]. These values indicate that AR interventions are highly effective for learning in individuals with ASD, and results suggest that AR could be a promising technology to learn skills in educational context following the quality indicators of Reichow [23].

The two studies that met the quality criteria to be classified as “strong” offered evidence on the integration of AR technology on teaching and learning process in autistic individuals [28,32]. Both studies taught a learning skill (i.e., knowledge of new science vocabulary terms and reaction time and executive functions). One of the studies that meet the quality criteria allowed ASD subjects to create their own AR experiences by matching trigger images/objects with user-created digital content that can include images and video [28]. This application is one of the most obvious benefits of AR applications for learning new vocabulary. This study expands mobile learning by integrating AR technology, allowing students to access augmented contextual information seamlessly within their physical environment. The findings highlight how AR and mobile devices facilitate a dynamic, adaptable learning experience, and reduce the overall dependency on a teacher. This might also facilitate the scaling of interventions with reduced resources, an increasingly important goal for low-resource settings in educational contexts. The other study that met the quality criteria [32] applied AR game-based cognitive–motor training, three core EFs (working memory, cognitive flexibility, and cognitive inhibition), and reaction time, which were measured at baseline and after the intervention. Findings indicated that, after four weeks of training, cognitive–motor exercises using AR-enhanced games effectively reduced stereotypic behaviors, compulsiveness, sameness, and restricted behaviors. Additionally, participants exhibited enhanced cognitive abilities, including improvements in working memory, cognitive flexibility, cognitive inhibition, reaction time, and attention. These results suggested that AR-based game interventions served as an effective tool for targeting both cognitive and motor functions in individuals with ASD in line with the previous literature [38].

Moreover, seven were classified as “adequate” and could offer some evidence on the integration of AR technology for learning different skills [26,27,29,30,34,35,36]. In all of them, AR improved the learning of several concepts and processes (i.e., learning science concepts/naming occupations, reading words and syllables, improving reaction time and joint attention in educational tasks, and learning mathematical concepts and problem-solving skills). Findings suggest that AR can simulate learning processes in a child, adolescent, or adult with ASD and are consistent with the results of previous review studies [18,22]. This review provides evidence that AR offers significant advantages in education, particularly for individuals with ASD, by supporting concept acquisition, reading development, and cognitive skills. These interactive technologies foster increased motivation, communication, and participation, making learning a more engaging experience [22].

Beyond academic learning, the review found that AR interventions positively impacted executive functions (EFs) and attentional skills in individuals with ASD. Studies by Antão et al. [30] and Nekar et al. [32] demonstrated improvements in working memory, cognitive flexibility, cognitive inhibition, and reaction time following AR-based cognitive–motor training. These findings align with the existing literature suggesting that digital interventions incorporating cognitive–motor tasks can enhance EFs in ASD populations. Similarly, attention-based AR interventions [33,34] were associated with increased on-task behavior and improved joint attention, reinforcing AR’s effectiveness in fostering engagement and attentional control.

However, in the current review, only one of the group experimental designs tested for generalization, and just a few of the single-case studies collected generalization data post-intervention. Unfortunately, this trend is consistent with other reviews [39]. Notably, more methodologically rigorous research is needed to determine the evidence for using AR for academic skills before it can be more widely implemented in the education of students with ASD [10,22]. Future research should evaluate generalization to the real world environment and evaluate if interventions facilitated via AR result in the improved generalization of academic skills.

### Limitations

Despite the promising findings, this review identified several methodological limitations within the included studies. Notably, the majority (n = 9) utilized single-subject designs, which restrict the generalizability of the results. While these studies offer valuable insights into individual progress, larger-scale investigations incorporating control groups are essential to establish the broader efficacy of AR interventions across diverse ASD populations. A key limitation of this review is the predominance of single-subject methodologies, which also precluded the presentation of effect size measurements due to the inherent constraints of the included studies. Future research should prioritize conducting meta-analyses that integrate effect size calculations and advanced statistical modeling to facilitate a more rigorous quantitative evaluation of the effectiveness of AR-based interventions. Furthermore, only one study [32] implemented a randomized controlled trial (RCT), underscoring the need for more rigorous experimental designs to strengthen the evidence base.

Another notable limitation was the participant demographics. The total sample of 123 individuals with ASD exhibited a significant gender imbalance, with only 11.88% of participants being female. While this distribution aligns with the expected male-to-female ratio in ASD diagnoses [37], the limited inclusion of female participants restricts the applicability of findings across genders. Additionally, most studies focused on children and adolescents, with only two studies including adults with ASD [30,35], highlighting the need for further research on AR interventions tailored to adult learners.

Intellectual functioning was another factor contributing to study limitations. While fifteen studies assessed individuals with an IQ > 70, only two studies [31,33] analyzed interventions in individuals with lower IQ scores (<70). This discrepancy limits the conclusions that can be drawn about AR’s effectiveness in individuals with varying cognitive abilities, emphasizing the necessity of future studies incorporating diverse cognitive profiles.

In terms of technology, a majority of studies (58.4%) utilized mobile AR applications, while the remaining 41.6% relied on desktop or tablet-based interventions. Although mobile AR platforms offer greater accessibility and flexibility, desktop-based applications may provide a more controlled and immersive learning environment. Future studies should explore the comparative advantages of these different technological approaches to determine optimal intervention strategies. Finaly, this review only included peer reviewed studies excluding a number of research in gray literature or conference papers, and only studies published in English were included. This language restriction may have led to the exclusion of relevant studies published in other languages, potentially limiting the comprehensiveness of the findings.

## 5. Conclusions

The integration of AR applications in educational contexts has demonstrated potential benefits for the learning process. The reviewed studies reported positive outcomes across various learning domains through the use of AR technology. While further research is needed, particularly with more robust methodologies and larger sample sizes, the current evidence suggests that AR-based interventions can support the development of both academic and cognitive skills in individuals with ASD. Most existing studies remain preliminary investigations or focus on technological development rather than controlled intervention trials, highlighting the need for further high-quality research.

This review enhances our understanding of AR-based interventions and their effectiveness in improving learning and cognitive skills in children, adolescents, and adults with ASD. It is crucial to continue investigating these interventions with well-designed scientific studies that assess their impact under controlled conditions. Furthermore, professionals should critically evaluate AR-based interventions to ensure that they are supported by evidence and appropriately tailored to the specific needs of individuals with ASD. While the findings are promising, continued research employing rigorous methodologies is necessary to strengthen the evidence base for AR as a valuable educational tool.

## Figures and Tables

**Table 1:** Main characteristics of the selected studies (N = 12).

Country	Author/Year	Participants: N, Age, % Males, IQ	Study Design	AR Technology/Evaluation	Dependent Variables	Main Results
Turkey	Denizli-Gulboy et al. [26]	ASD (4), 10–13.6 years, 100% males, IQ (>70)	Single-subject multiple baseline design, multiple probe design	‘Vucudumuz 4D’ AR mobile application/AR cards, tablet computer	Learning science concepts	AR application was effective in naming the internal organs. AR application improved the non-target skill of explaining organ function
Turkey	Gulboy et al. [27]	ASD (3), 3–5 years, 100% males, IQ (>70)	Single-subject multiple baseline design	Octaland 4D AR application to teach the occupations	Learning naming occupations	AR application was effective in teaching occupation (i.e., “Architect”) and non-targeted information (i.e., “Design building”)
Malaysia	Hashim et al. [16]	ASD (6), 5–12 years, 83% males, IQ (>70)	Single-subject multiple baseline design/qualitative aproach	AR smartphone application, ‘AReal-Vocab’	Learning English vocabulary	AR application was successful in stimulating pronunciation abilities and language articulation
USA	McMahon et al. [28]	ASD (1) Male 25 years, IQ > 70 ID (3) (75% female), 19–25 years, IQ low	Single-subject multiple baseline design	Aurasma application, mobile device with AR content	Learning science vocabulary words	All students acquired definition and labeling knowledge for the new science vocabulary terms.
Indonesia	Khoirunnisa et al. [29]	ASD (4), 8–13 years, 100% males, IQ (>70)	Single-subject design	Mobile AR application	Reading words with SAS method	The ability to read words and syllables experienced an increase. AR application was effective in supporting early reading learning for children with ASD
Brazil	Antão et al. [30]	ASD (48), 11 (5.0) mean age, TD (48), 11.8 (5.2) mean age, 89% males, IQ (>70)	Non-randomized group design	Use of alphabet letter and number in an AR task	Reaction time (RT)	Only the ASD group showed an improvement in the performance of the total RT after the AR task
Malaysia	Mahayuddin et al. [31]	ASD (10), 8–10 years, males and females, IQ (>70)	Within-subject design	AR mobile learning platform versus conventional teaching	Learning of phonics, literacy, and spelling words	AR mobile learning improved phonics learning and children could capture and associate the graphics or pictures of the surrounding objects
Korea	Nekar et al. [32]	ASD (24) 6–18 years, 91.6% males, IQ (>70) 12 experimental groups, 12 control groups	Pretest–posttest design	AR game-based cognitive training	EF and RT	Significant improvements in RT and EF (working memory, cognitive flexibility, and cognitive inhibition) in the experimental group
Mexico	Escobedo et al. [33]	ASD (12), 3–8 years, IQ (>70), n.r. gender	Single-subject design	Mobis AR application	Selective and sustained attention	Mobis increased the time students remained on task by 20 percent AR application increases selective and sustained attention
Spain	Perez et al. [34]	ASD (6), 3–9 years, 83% males, IQ low	Single-subject design	AR Technology- mediated intervention (Pictogram room)	Learning body knowledge, imitation, joint attention skills	All children improved performance in joint attention skills (following the gaze of a dummy and pointing to the object that the dummy was looking at) following the intervention generalized to real-world situations
USA	Root et al. [35]	ASD (4), 21 years, 50% males, IQ (>70)	Single-case multiple probe design	Video-based instruction via AR	Learning mathematical problem-solving skills	Significant relation between the intervention and personal finance problem- solving skills. Participants were also able to self-correct errors after watching AR-triggered model videos
USA	Morris et al. [36]	ASD (1), 15 years, female, IQ (>70), LD (1), 14 years, Female, IQ (>70)	Single-subject design	Point-of-view video modeling with AR app	Learning mathematics	Improvement in mathematics performance (i.e., adding fractions with common denominators) Participants demonstrated high levels of maintenance and were able to apply the skills to word problems without additional training

ASD: autism spectrum disorder; AR: augmented reality; n.r.: not reported; EF: executive function; N: number; IQ: intelligence quotient; RT: reaction time; TD: typical development; ID: intellectual disability; SAS: synthetic structural analytical; and LD: learning disability.

**Table 2:** Learning outcomes for included studies.

Reference	Science	Language/ Vocabulary	Reading	Reaction Time/EF	Attention Skills	Mathematics
Denizli-Gulboy et al. [26]	X					
Gulboy et al. [27]		X				
Hashim et al. [16]		X				
McMahon et al. [28]	X					
Khoirunnisa et al. [29]			X			
Antão et al. [30]				X		
Mahayuddin et al. [31]			X			
Nekar et al. [32]				X		
Escobedo et al. [33]					X	
Perez et al. [34]					X	
Root et al. [35]						X
Morris et al. [36]						X

**Table 3:** Summary of the strength of each study.

Reference	Strength Rating (Reichow [23])
Denizli-Gulboy et al. [26]	Adequate
Gulboy et al. [27]	Adequate
Hashim et al. [16]	Weak
McMahon et al. [28]	Strong
Khoirunnisa et al. [29]	Adequate
Antão et al. [30]	Adequate
Mahayuddin et al. [31]	Weak
Nekar et al. [32]	Strong
Escobedo et al. [33]	Weak
Perez et al. [34]	Adequate
Root et al. [35]	Adequate
Morris et al. [36]	Adequate

## Data Availability

The original contributions presented in this study are included in the article. Further inquiries can be directed to the corresponding author.
